# Prospects for using plant-based biomass in the construction of bio-based houses

**DOI:** 10.3389/fpls.2025.1697154

**Published:** 2025-10-14

**Authors:** Francesco Pancaldi, Martien van den Oever, Rommie van der Weide, Sven van Baren, Sanabel Abdulbawab, Sabine van Rooij, Michael van Buuren, Marcel van der Voort, Arjen van Kampen, Luisa M. Trindade

**Affiliations:** ^1^ Plant Breeding, Wageningen University & Research, Wageningen, Netherlands; ^2^ Wageningen Food & Biobased Research, Wageningen University & Research, Wageningen, Netherlands; ^3^ Wageningen Plant Research, Lelystad, Netherlands; ^4^ Wageningen Environmental Research, Wageningen, Netherlands; ^5^ Faculty of Textiles, Engineering and Business, University of Boras, Boras, Sweden

**Keywords:** biomaterials, lignocellulosic crops, biobased houses, biomaterial value chains, fibre crops, woody crops, biobased construction

## Abstract

The construction industry is a major contributor to climate change, due to the extensive use of non-renewable materials, such as concrete and steel. Bio-based materials manufactured from diverse plant biomass sources – mainly wood, lignocellulosic biomass, and plant fibres – offer sustainable alternatives, potentially transforming buildings into net carbon sinks. However, the establishment of effective value chains for the provision and deployment of biomass in “largely bio-based” houses (i.e. houses with main elements made up of bio-based materials) is still far from being reached. This depends largely on the level of optimisation of bio-based vs conventional construction materials. In this context, this opinion paper explores the feasibility of building “largely bio-based” houses by discussing both the availability and the diverse functional roles that different biomass types from diverse plant species can have in construction applications. Moreover, the article highlights current research challenges in the supply of high-quality biomass for “bio-based houses”. Finally, it discusses how the effective integration of plant science, material engineering, as well as environmental and economic research in trans-disciplinary research efforts is key to set up operational and self-standing bio-based construction value chains.

## 1 Introduction

The global construction industry causes extensive negative environmental impacts and represents a major driver of climate change. On the one hand, this depends on the enormous use of concrete, steel, aluminium, and glass. These materials are manufactured by using large amounts of non-renewable resources, such as iron, bauxite, limestone, clay, sand, and rock aggregates, whose extraction and processing causes water pollution, destruction of natural habitats, deforestation, and soil erosion (Gavriletea, 2017; Joshi et al., 2022; Silveira et al., 2021; Pradhan et al., 2024). On the other hand, the construction sector consumes vast amounts of energy across value chains – from production of construction materials, to their global trading, to the construction of buildings. As such, the construction sector currently accounts for 10-15% of the annual global greenhouse gas emissions (UNEP, 2022, 2023).

Actions to mitigate the environmental and climate impact of the global construction industry are urgently needed, especially in view of the global demographic and urbanization dynamics (UNEP, 2023; Marinova et al., 2020; Deetman et al., 2020). In fact, both population growth and urbanization are expanding global demand for new buildings, which is expected to grow ~50% over 2020 levels by 2050 (Deetman et al., 2020). In turn, increased housing demands will also expand the global demand for construction materials, with a potential deterioration of the associated impacts on environment and climate (Marinova et al., 2020; Deetman et al., 2020). In this context, bio-based materials can offer options to (partly) replace non-renewable construction materials and improve the environmental and carbon footprints of the global construction industry, possibly turning buildings into net carbon sinks (Crawford and Cadorel, 2017; Churkina et al., 2020; Abed et al., 2022). However, there is still unclarity about best practices in the deployment of bio-based materials in constructions, to promote sustainability in the sector while avoiding negative side-effects on land use, biodiversity, carbon cycles, and prices of construction materials and houses.

Recent research on deploying bio-based materials in construction focused mainly on the use of mass timber (primarily cross-laminated timber – CLT – and glue laminated timber – Glulam) in buildings to replace steel and concrete in load-bearing elements, highlighting good potential (Crawford and Cadorel, 2017; Churkina et al., 2020; Abed et al., 2022). However, next to mass timber, numerous other bio-based alternatives can be used to produce other relevant construction materials, including particles and fibres from lignocellulosic crops and by-products of agricultural practices. Taking an integral perspective on the construction value chains, by considering all the bioresources that can be used to build “largely bio-based houses” (i.e. houses with main elements made up of bio-based materials) is pivotal to establish a bio-based construction sector where biomass functionality is aligned with biomass productivity, at the same time ensuring overall system sustainability. In this view, this *perspective paper* aims at providing an overview of promising biomass sources for building “largely bio-based houses”, with a discussion on their physical properties, environmental impact, biomass availability, and socio-economic aspects. Moreover, avenues for future agricultural and engineering research are also discussed, with the goal of exploring best practices and challenges for shifting the construction sector toward sustainable and circular models.

## 2 Different biomass sources for different house components

**Figure 1:**
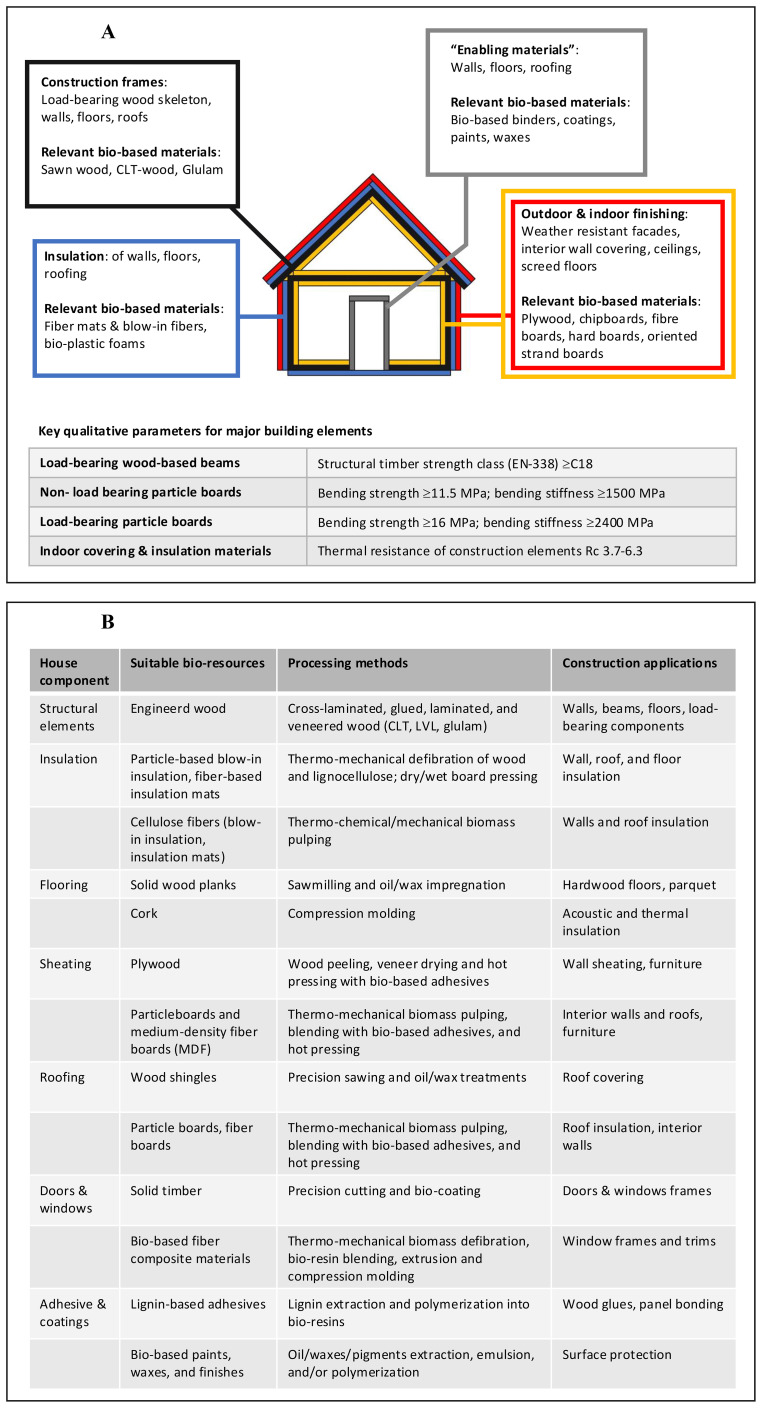
**(A)** Main structural and finishing construction elements found in a house, potential bio-based materials that can potentially be used for their manufacturing, and relevant qualitative parameters that deployed materials need to meet; **(B)** Classes of bioresources that can be used to manufacture bio-based materials for different construction needs in bio-based houses, with indication of methods for their manufacturing and construction applications.

Central to the design of bio-based houses is the structural complexity of buildings, which combine different elements requiring specific properties, particularly in terms of mechanical characteristics, physical structure and behaviour, durability, and visual appearance (**Figure 1A**). Therefore, material choice is critical to correctly design bio-based houses, and the materials used must satisfy the properties required by different building parts. In this regard, different classes of bioresources appear most suitable for different elements of a bio-based house (**Figure 1B**). Specifically, sawn wood, glulam, and cross laminated timber are preferred materials to be deployed in the construction framework of a bio-based house, thanks to their high bending strength and stiffness (He et al., 2018; Arriaga et al., 2023; Ilgın, 2023). In parallel, wood-based panels and particle boards made up of lignocellulose aggregates are ideal for semi-structural elements as inner walls, given their relatively light weight combined with acceptable stiffness and density, as well as low costs (Klímek et al., 2018; Martins et al., 2021; Moll et al., 2024). Conversely, plant-based fibres converted into low-density mats or panels are ideal for use in insulation layers, even if attention should be paid for their durability and ignition resistance (Charai et al., 2021; Chihab et al., 2023).

While it is therefore clear that effective bio-based alternatives of conventional construction materials exist, open questions remain about both the sustainable sourcing of these bioresources from current agricultural landscapes, and the research needed to optimize these bioresources from a whole value chain point of view. In this regard, an important aspect entails the identification of best plant species, crops, and/or crop residues that can supply the timber, particles (chips), fibres, and other materials of **Figure 1B**, in good amounts to satisfy their deployment in economies of scale, while considering environmental and economic sustainability. Moreover, identifying bottlenecks for cost- and quality-effective biomass production is also important to enable this vision.

## 3 Promising crops and cropping systems to deliver biomass sources for the construction sector: a comparative analysis

Several plant species and crops can be used to supply suitable biomass for building the different parts of “bio-based houses” (**Table 1**). However, the optimal allocation of biomass crops within multi-purpose agricultural systems must consider several factors, balancing out production characteristics (quantity and composition of the biomass) against suitability of plant species and varieties for local soil, water and climate conditions. This aspect is particularly important for selecting trees for wood supply, as the natural growing area of tree species for timber production determines geographical patterns of wood production (Verkerk et al., 2015, 2019). Therefore, an accurate selection of tree species for wood supply to construction is pivotal, especially when promoting locally-oriented value chains for biomass supply is also a goal. This is a key aspect, as local biomass sourcing minimizes the carbon footprint of bio-based houses, while also promoting rural development through inter-sectorial value chains (agriculture, wood processing, construction industries) (Palander and Vesa, 2022; Dams et al., 2023).

**Table 1:** Plant species suitable for building the different parts of bio-based houses.

Plant species	Common name	Species type	Dry wood/biomass yield ranges across EU environments	Time until production maturity (years, unless otherwise specified)	Construction applications	References
*Fagus sylvatica*	Beech	Hardwood tree	1–2 t/ha/year	80-100	Structural elements, interior panelling, flooring, furniture	(Sell, 1987; Pretzsch et al., 2014)
*Betula pendula*	Silver birch	Hardwood tree	1–4 t/ha/year	40-50	Plywood, flooring, interior fittings	(Johansson, 1999; Rowell, 2005)
*Quercus* spp.	Oak	Hardwood tree	1–4 t/ha/year	80-120	Structural beams, flooring, furniture	(Jones, 1959; Sánchez-Gonzáleza et al., 2005)
*Picea abies*	Norway spruce	Softwood tree	5–15 t/ha/year	60-80	Structural walls and beams, roof trusses, windows and doors frames	(Kollmann and Côté, 1968; Pretzsch, 2009)
*Pinus sylvestris*	Scots pine	Softwood tree	5–15 t/ha/year	60-100	Structural elements, exterior cladding, treated wood, plywood	(Rydholm, 1965; Hynynen et al., 2002; Zianis et al., 2005)
*Populus* spp.	Poplar	Softwood tree	7–13 t/ha/year	2-10 (short rotation regime); 15-30 (medium/long rotation regimes)	Lightweight plywood, OSB for walls and flooring, composite materials	(Scarascia-Mugnozza et al., 1997; Kauter et al., 2003)
*Miscanthus* spp.	Miscanthus	Lignocellulosic crop	12–30 t/ha/year	2–3 years until crop maturity, followed by annual harvests	Fibreboards and particleboards, insulation panels	(Van Der Weijde et al., 2013; Tajuddin et al., 2016; Klímek et al., 2018)
*Panicum virgatum*	Switchgrass	Lignocellulosic crop	9–22 t/ha/year	2–3 years until crop maturity, followed by annual harvests	Composite boards, insulation panels, exterior cladding	(Van Der Weijde et al., 2013; Janiszewska et al., 2022; Martyniak et al., 2025)
*Cannabis sativa*	Hemp	Lignocellulose/fibre crop	5–15 t/ha/year	3–5 months	Bio-based panels, insulation panels, fibre boards	(Jami et al., 2022; Martínez et al., 2023; Pancaldi et al., 2025)
*Linum usitatissimum*	Flax	Lignocellulose/fiber crop	1.5–3 t/ha/year	3–5 months	Fiber boards, composite elements	(Pisupati et al., 2021; Arslanoglu et al., 2022; Mohamed et al., 2025)

Considering the aspects just discussed, relatively abundant sources of hard wood that can overall target different geographical ranges are beech (*Fagus sylvatica*), silver birch (*Betula pendula*), and different oak species (*Quercus* spp.) (**Table 1**). Conversely, attractive soft wood species include Norway spruce (*Picea abies*), Scots pine (*Pinus sylvestris*) and poplar trees (*Populus* spp.). Overall, these tree species grow on relatively large acreages in Europe and can be sustainably cultivated under regimes of (short) rotation forestry, as well as sourced through sustainable management of natural forests (Walle et al., 2007; Lundmark et al., 2018; Ekholm et al., 2023). These types of agronomic practices promote prolonged land coverage, increasing the soil, plant, and animal biodiversity of cropping systems (Ekholm et al., 2023; Oliveira et al., 2024). Moreover, silviculture represents a promising activity to prevent abandonment of fragile environments, such as European mountain areas (Dax et al., 2021). For all these practices, the duration of the rotation cycles of tree cultivation and of tree harvesting from natural forests is a critical factor to modulate the hardness and density of wood, through the molecular regulation of plant cell walls deposition (i.e., thickness of plant cell walls and total amount of cellulose and lignin) (Guidi et al., 2009; Rizanti et al., 2018; Ding et al., 2022). These aspects are particularly important to meet specific wood quality standards for construction applications, as the molecular composition of wood determines resistance to mechanical stress and water damage, stiffness, and durability (Toumpanaki et al., 2021; Ding et al., 2022). Additionally, the length of forestry rotations and frequency of tree harvesting significantly affects the economic profitability of wood production (Chen et al., 2017). Therefore, species- and end-use-specific choices in crop systems management, along with a transversal minimization of agricultural inputs, are key to establish profitable value chains. Moreover, an effective allocation of wood cropping systems into agricultural landscapes is also key. As such, while converting fertile land to the cultivation of trees for biomass production can lead to loss of profits for farmers (especially when the benefits for land coverage and biodiversity preservation are not subsidized), the allocation of tree production to marginal lands (i.e. environmentally-degraded lands currently not used by agriculture) can create new income sources in specific regions (Abolina and Luzadis, 2015; Valujeva et al., 2022). Recent studies indicated that about 30 Mha of marginal lands are available at the European level (Von Cossel et al., 2019). This area could potentially sustain a standing wood stock of ~5.5 billion m^3^, assuming a tree density in line with European natural forests, and mid-aged trees (Pilli et al., 2023).

Next to trees, several lignocellulosic and/or fibre crops also represent very important species to provide biomass and plant-based fibres for building bio-based houses, especially for manufacturing particle boards, insulation elements, and textiles. These crops include species as *Miscanthus* spp., switchgrass (*Panicum virgatum*), willow (*Salix* spp.), hemp (*Cannabis sativa*), flax (*Linum usitatissimum*), and nettles (*Urtica dioica*) (**Table 1**). As discussed for trees, the allocation of these crops to agricultural systems should carefully consider the performance of different species under different environmental conditions, as the growing environment can heavily affect the biomass yield and quality of these crops (Fabio et al., 2017; Petit et al., 2020; Awty-Carroll et al., 2023). On the one hand, maximizing biomass yield is pivotal for achieving a profitable cultivation of these species. On the other hand, biomass quality – generally meant as an optimal ratio of molecular components of the plant cell walls toward specific applications – is key to ensure a technically- and cost-effective processing of the biomass into final applications (Pancaldi and Trindade, 2020; Van Der Cruijsen et al., 2021, Goudenhooft et al., 2019). Overall, crops as Miscanthus, switchgrass, hemp, flax, nettles, and willow can supply lignocellulose and fibres for particle boards and insulation materials, but so far these feedstock do not reach P5 quality (international standards for particle boards) (van den Oever et al., 2024). This is mainly due to their cell wall properties – high silica and wax content, and thin, porous structures – that reduce resin bonding efficiency and increase moisture uptake, preventing compliance with the mechanical strength and humidity-resistance requirements of P5 (van den Oever et al., 2024). Regarding growing conditions, fibre crops display have advantages over tree species in terms of growing speed and adaptation to a wide range of environments, including cooler and warmer locations across large latitudinal ranges (Petit et al., 2020; Awty-Carroll et al., 2023). Moreover, these crops can be introduced within the agricultural systems in different ways, while it is more challenging to introduce trees in agricultural landscapes. For example, annual fibre crops as hemp or flax can act as break crops in rotation with cereals and several annual staple crops (potatoes, sugar beet and some oilseeds). By contrast, perennial species as Miscanthus, switchgrass and nettles can be included into current agricultural landscapes under strip cropping regimes with food crops or, as discussed for woody species, through cultivation on marginal lands. Under this latter scenario, European marginal lands could potentially produce ~240 Mt of biomass per year [considering an average biomass yield of ~8 t/ha] (Nijsen et al., 2012). In parallel, when the cultivation of perennial biomass crops on marginal lands would take place through the establishment of mixed cropping systems with multiple species, the presence of these crops can promote synergy between the use of agricultural resources and increasing biodiversity, ecosystem services, and soil quality of marginal lands (Carlsson et al., 2017; Pancaldi and Trindade, 2020). Nevertheless, the establishment of subsidies for biodiversity benefits through these types of cropping systems and for sustainable biomass use are still critical factors to boost such value chains.

## 4 Research bottlenecks in the optimization of bioresources for the construction sector

The previous section highlighted the critical role of selecting appropriate crops and cropping systems to sustainably supply biomass for building bio-based houses. However, the development of sustainable and economically-competitive value chains, from biomass sourcing until deployment of bio-based products in constructions, extends beyond decisions on agronomic practices and agricultural planning. Specifically, both the transformation of bioresources into finished products and the development of crops able to withstand growing conditions found on lands not used for food production (while maintaining good biomass quality) pose challenges to plant scientists, as well as process and material engineers. In fact, the inherent molecular makeup of plant biomass, which strongly depends on the genetics and biology of biomass crops, is a major driver of the costs to transform such biomass into bio-based products (Pancaldi and Trindade, 2020; Van Der Cruijsen et al., 2021). This is because the relative proportions of molecular components of lignocellulosic biomass, such as lignin, cellulose, and hemicellulose, along with the content of ash and of secondary metabolites, play a crucial role in the mechanical, thermal, and chemical processes required for biomass conversion into construction products (Pancaldi and Trindade, 2020; Van Der Cruijsen et al., 2021). Consequently, the efficiency, scalability, and affordability of biomass conversion routes are also affected. A further level of complexity is represented by the relative novelty of plant molecular targets underlying the optimal processing of biomass into construction products, as well as of the technical processing routes. Therefore, we envision that integration of research efforts between plant scientists and engineers, with the aim of both improving the genetic makeup of crops that controls biomass quality and to optimize cost-effective processing of biomass into construction products, will be pivotal to reduce costs of bio-based construction materials, while ensuring sustainability of biomass production and processing for construction.

A concrete case showing how plant science and engineering expertise can be effectively leveraged into research lines that address the complete value chains of bio-based construction materials is given by the transformation of Miscanthus biomass into different final products: particle boards, insulation panels, viscose for textiles, and construction chemicals (e.g. adhesives). Once harvested and dried, Miscanthus biomass is composed for 80-90% w/w by plant cell walls, and for the remaining part essentially by ash (Van Der Weijde et al., 2017a; Xu et al., 2020; Pancaldi et al., 2023). Depending on the desired final products, the dried biomass is typically chopped and subsequently thermally or chemically treated, with the aim of either exposing lignin and hemicellulose to improve chips binding into boards, or of purifying cellulose and lignin for manufacturing viscose fibres and natural adhesives (Klímek et al., 2018; Park et al., 2012; Liu et al., 2022; Götz et al., 2022). For all these purposes, the content, branching and molecular organization of lignin, cellulose, and hemicellulose within plant cell walls significantly affect both the cost of thermo-chemical biomass treatments and the yield of fibres and chemicals. Therefore, plant breeding and biotechnology strategies could be applied to develop fit-for-purpose Miscanthus varieties. For example, by manipulating genes underlying cellulose, hemicellulose, and lignin synthesis, level of cellulose crystallinity, amount of hemicellulose substitutions, and ratio of different monolignols within biomass (Torres et al., 2015; Van Der Weijde et al., 2017b, Pancaldi et al., 2023). This way, fit-for-purpose varieties with minimized ash content, higher cellulose and lignin content, and modified bonding between lignin, cellulose and hemicellulose could be developed, to favour the production of the different potential outlets: particle boards, insulation panels, viscose fibres, bio-adhesives, etc. In parallel, process engineers could optimize milder and cheaper treatments for optimized Miscanthus varieties, whose molecular biomass structure inherently favours the processing into specific products.

The trans-disciplinary research approach just proposed could be extended even beyond plant science and process engineering, embracing environmental research and logistics engineering. The aims would consist in precisely quantifying the amount of biomass with specific properties needed to satisfy the housing demand in specific regions, to model optimal scenarios in the allocation of crop varieties within agricultural systems to satisfy such demand, and to calculate carbon savings of bio-based construction value chains. In this regard, data from architectural prototypes indicate that building bio-based houses can require ~13 tons of wood (for a ~100 m^2^ house) (Ballinas and Chávez, 2023) or ~4 tons of miscanthus straw (for a ~40 m^2^ house where outer walls are built with miscanthus straw) (Thornton et al., 2025). Assuming an average building lifetime of 50 years, this material use translates to a land pressure of ~0.08 ha/year for the wood case and ~0.007 ha/year for the miscanthus case (assuming yields of 6 and 12 t/ha for forest timber and miscanthus, respectively – see **Table 1**). These estimates could be crossed with other types of data, including geo-spatial data about land use, or information on crop phenology and yield of diverse crop varieties. This way, a spatial assessment of the boundaries for manufacturing bio-based houses with biomass sourced from specific regions could be obtained. In turn, this type of information at local scale would allow to plan bio-based construction value chains within specific territories and across traditionally not-connected sectors.

## 5 Outlook

Building bio-based houses represents a great opportunity to promote sustainability, circularity, and carbon-storage practices in the global construction sector, which has currently significant impact on climate change. Recent studies suggest great potential for producing bio-based construction materials by using biomass from trees, industrial crops, and agricultural side streams (Daly and Barril, 2024). However, this vision poses also important challenges ahead. Specifically, given the finite amount of land and forests and a growing housing demand, it is pivotal to coordinate research efforts to carefully model the most effective ways of sustainably allocating biomass sources to meet construction demands. Moreover, improvement of crops to maximize both biomass production and biomass quality to manufacture specific construction products is also critical. In parallel, optimization of biomass processing based on biomass properties and construction needs is equally relevant. Overall, we firmly believe that research lines that combine all these aspects in unitary, trans-disciplinary, whole value-chain research efforts represent the most effective way to reach the goals above. Moreover, this vision allows to also directly scale research ideas into real-world value chains, by involving industrial partners and regulatory institutions along the process. This is crucial, as scalability is key to make value chains economically independent. The solution of this bottleneck will likely mark the starting point of implementation bio-based houses at large scales.

## Data Availability

The original contributions presented in the study are included in the article/supplementary material. Further inquiries can be directed to the corresponding author.
